# Intrabronchial schwannoma with right lower lobe obstructive bronchiectasis and organizing pneumonia: A case report

**DOI:** 10.1002/rcr2.70044

**Published:** 2024-10-07

**Authors:** Bo‐Hsiang Lu, You‐Cheng Jiang, Jang‐Shian Liang, Ping‐Chung Tsai, En‐Kuei Tang

**Affiliations:** ^1^ Division of Thoracic Surgery, Department of Surgery Kaohsiung Veterans General Hospital Kaohsiung Taiwan; ^2^ Division of Chest Medicine, Department of Internal Medicine Kaohsiung Veterans General Hospital Kaohsiung Taiwan; ^3^ Department of Pathology and Laboratory Medicine Kaohsiung Veterans General Hospital Kaohsiung Taiwan; ^4^ Institute of Emergency and Critical Care Medicine, School of Medicine National Yang Ming Chiao Tung University Taipei Taiwan

**Keywords:** bronchial reconstruction, intrabronchial schwannoma, obstructive pneumonitis

## Abstract

We present the case of a 60‐year‐old female patient with no prior history of any systemic disease. She suffered from a prolonged cough that lasted more than 3 months, associated with poor appetite and weight loss of 5 kg. The pathology report of the pre‐operative transbronchial needle biopsy was consistent with a neurogenic tumour. Chest computed tomography (CT) revealed a right lower lobe (RLL) mass‐like consolidation of 8.67 cm with obstructive pneumonitis and suspicious posterior mediastinal invasion. The tumour was surgically resected with bronchial reconstruction, and the pathological diagnosis was intrabronchial schwannoma located inside the bronchus, a rare tumour that should be included as one of the differential diagnoses of primary bronchial tumours. The possibility of a surgical completed resection should be considered in patients with airway obstruction symptoms.

## Graphical abstract

We present the management of a rare case of intrabronchial schwannoma located inside the bronchus.

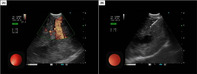

## INTRODUCTION

Schwannoma is a benign encapsulated tumour of the peripheral nervous system, that forms lesions within the thoracic cavity more typically seen in the posterior mediastinum, paraspinal regions, or ribs.^1^ Although one‐quarter of pulmonary neurogenic tumours are located in the trachea or bronchus,^1^ schwannomas rarely develop in the lung. Airway lesions may be asymptomatic and are accidentally screened using imaging examinations or from symptoms related to airway narrowing or mucosal irritation. Clinical presentation depends on the size and location of the tumour, and extent of obstruction. Surgical resection remains the most common treatment for diagnosed intrabronchial schwannomas with an excellent prognosis, while endoscopic treatments can also be effective and should be considered in selected patients.^2^

## CASE REPORT

**FIGURE 1:**
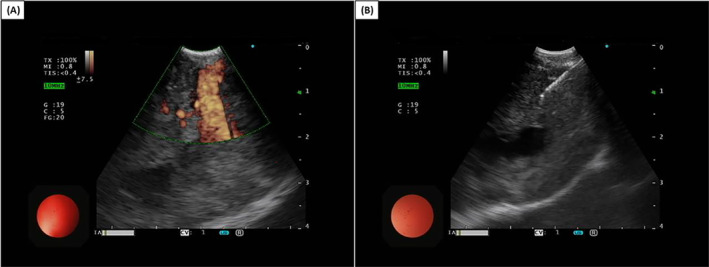
(A) An enlarged intrabronchial tumour under endobronchial ultrasound and (B) transbronchial needle aspiration biopsy.

**FIGURE 2:**
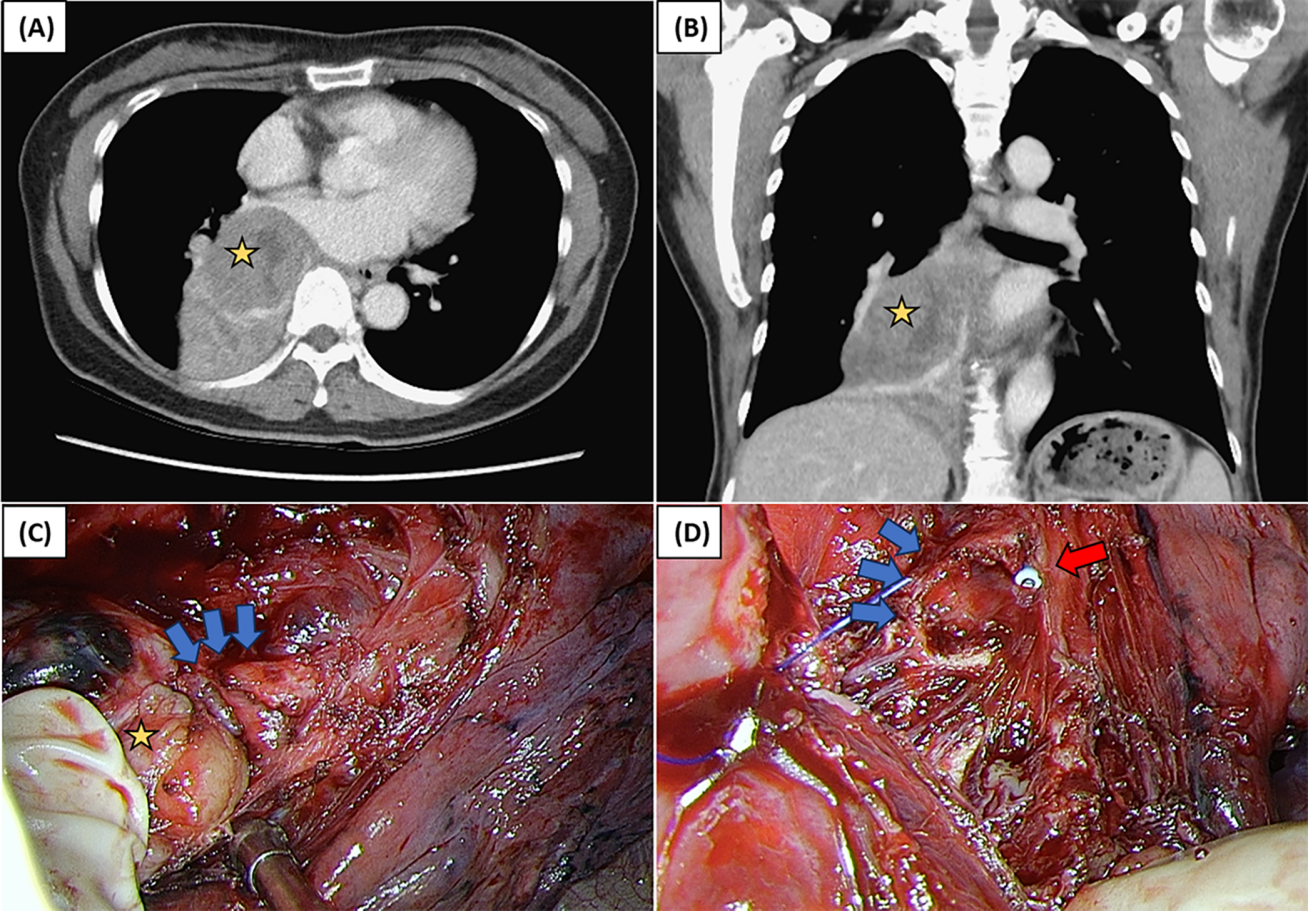
(A) Contrast enhanced computed tomography (CT) axis view: Intra‐bronchial schwannoma (yellow star) with distal obstruction of the right lower lobe. (B) CT coronal view of intra‐bronchial schwannoma (yellow star). (C) Intraoperatively, the tumour (yellow star) was pulled out from the bronchus intermedius (blue arrow). (D) After removal of the tumour, bronchus intermedius (blue arrow) with distal bronchiectasis and the tip of endotracheal blocker (red arrow) were seen.

A 60‐year‐old female with total independence in activities of daily living and no diagnosed underlying diseases suffered from a prolonged chronic cough lasting more than 3 months, which was exacerbated while lying down and associated with poor appetite and body weight loss of approximately 5 kg. In March 2024, a medial right lower lobe (RLL) central necrotic mass with obstructive pneumonitis and suspicious posterior mediastinal invasion was reported in a chest computed tomography (CT). Subsequently, several examinations such as bronchoscopy biopsy, transbronchial needle aspiration biopsy (Figure 1), or rigid bronchoscopy for tissue proof confirmed the pathologic report of neurogenic tumour (Ki‐67 labeling index up to 15%). However, the patient was transferred to our hospital for personal reasons where a repeat chest CT with contrast was arranged in June 2024. An 8.67 cm RLL mass with compression of right pulmonary vessels (inferior pulmonary vein, main pulmonary artery), right atrium, and bronchus intermedium were demonstrated in the axis (Figure 2A) and coronal (Figure 2B) CT scans.

**FIGURE 3:**
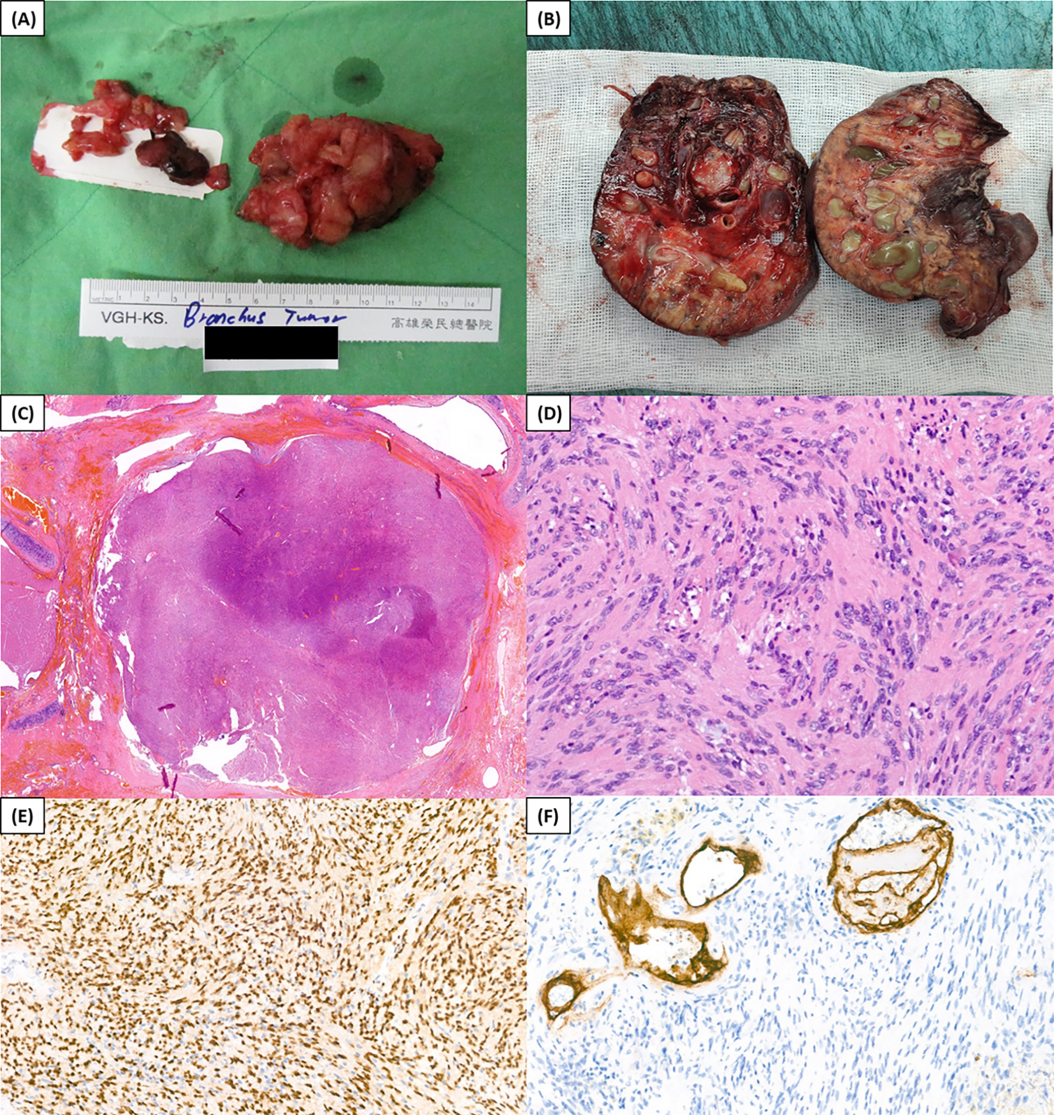
(A) Intrabronchial schwannoma. (B) Grossing section of the lung parenchyma with dilated bronchus (focally impacted with tumour) and distal bronchiectasis. (C and D) Tumour in bronchiole (haematoxylin and eosin staining, 20×). (E) Immunohistochemical staining for SOX10 (400×). (F) Immunohistochemical staining for CD34 (400×).

The patient underwent complete right posterolateral thoracotomy following thoracoscopic tumour resection. A yellowish homogeneous soft‐tissue mass was extracted from the bronchus intermedius (Figure 2C), entering the over‐extended bronchi. The blue tip of the endotracheal blocker was observed in the proximal bronchus intermedius (Figure 2D) and distal bronchiectasis of the bronchus after the tumour removal. Sleeve bronchial reconstruction was performed between the distant bronchus intermedius and right middle lobe orifice of the bronchus, then the pericardial fat pad flap was covered and reinforced for anastomosis. A large bronchial tumour (Figure 3A) was removed before surgical resection for irreversible lung parenchyma of RLL. The grossing section of the lung parenchyma demonstrated dilated bronchus with focal tumour impaction and distal bronchiectasis (Figure 3B). Microscopically, the haematoxylin and eosin staining (Figure 3C,D, respectively) showed a schwannoma composed of cellular spindle cells in dense palisading fashion (Antoni A area) and alternating loosely arranged area (Antoni B area), without marked cellular atypia, mitotic figure or necrosis. Immunohistochemically, the tumour cells were immunoreactive for SOX10 (Figure 3E) and S100, while negative for CD34 (Figure 3F), Desmin, H‐caldesmon, and SS18‐SSX. Further, H3K27me3 immunostain shows preserved expression.

## DISCUSSION

Trachea‐bronchial tumours constitute only 0.6% of respiratory tract neoplasms, a broad range of malignant and benign neoplasms with varying degrees of rarity. Among them, benign lesions account for only 10% of primary airway tumours in adults, mostly arising from mesenchymal tissue.^3^ These benign trachea‐bronchial tumours originated from the mesenchyma have neurogenic lesions (neurofibroma more often than schwannoma) manifested as well‐circumscribed rounded or lobulated homogeneous soft tissue nodules or masses with no distinguishing features.^1^ Generally, intrabronchial schwannoma is a rare entity, accounting for just 2% of benign tracheobronchial tumours.^4^ Aoyama et al.^4^ identified 33 cases of bronchial schwannoma, with a mean age of 48 years and female‐to‐male ratio of approximately 1:1. Of these cases, 15 were in the right bronchial tree, 15 were in the left bronchial tree, and 3 were in the trachea or carina. No apparent differences with regard to gender or tumour location were observed.

With the wide application of bronchoscopic treatment, high‐risk patients can select alternative options to surgery. Once endoscopists identify benign tumours of the tracheobronchial tree, bronchoscopic tumour removal or laser resection are considered the first line of therapy if the nature of the tumour is strictly endoluminal and limited to within the endobronchial tree.^2^ Further, schwannomas over endobronchial tree present a smooth and whitish vascular network on the surface under endoscopic appearance and favourable prognosis if easily resected when pedunculated. However, tumours with extraluminal involvement present a greater recurrence risk of bronchoscopically resected lesions.^5^ Thus, surgery may be necessary for larger lesions with difficulty in excluding potential malignancies, or peripheral destructive lung disease by long‐term atelectasis or obstructive pneumonia. Surgical resection is recommended followed by extrabronchial growth or dilemma in bronchoscopic procedures due to the multidirectional development of the tumour.^2^ Even though there is a low risk of recurrence after adequate treatment, bronchoscopy or image surveillance should be offered for early detection. Owing to the benign nature and slow growth characteristics of schwannomas, repeat endoscopic resection after recurrence may become a viable treatment option after recurrence.^5^

In conclusion, adequate treatment of intrabronchial schwannomas should consider all possible clinical conditions, despite the scarcity of other non‐diagnosed medical conditions. Surgical completed resection should be considered in patients who present with symptoms of airway obstruction. Long‐term bronchoscopic surveillance is necessary to prevent tumour recurrence.

## AUTHOR CONTRIBUTIONS

Bo‐Hsiang Lu and Ping‐Chung Tsai contributed substantially to writing the manuscript; You‐Cheng Jiang, Jang‐Shian Liang and En‐Kuei Tang contributed substantially to its critical review; All authors read and approved the final version of the manuscript.

## CONFLICT OF INTEREST STATEMENT

None declared.

## ETHICS STATEMENT

The authors declare that appropriate written informed consent was obtained for the publication of this manuscript and accompanying images.

## Data Availability

Data available on request from the authors.

## References

[1] Girvin F , Phan A , Steinberger S , Shostak E , Bessich J , Zhou F , et al. Malignant and benign tracheobronchial neoplasms: comprehensive review with radiologic, bronchoscopic, and pathologic correlation. Radiographics. 2023;43(9):e230045. 37561643 10.1148/rg.230045

[2] Shah H , Garbe L , Nussbaum E , Dumon JF , Chiodera PL , Cavaliere S . Benign tumors of the tracheobronchial tree. Endoscopic characteristics and role of laser resection. Chest. 1995;107(6):1744–1751. 7781378 10.1378/chest.107.6.1744

[3] Macchiarini P . Primary tracheal tumours. Lancet Oncol. 2006;7(1):83–91. 16389188 10.1016/S1470-2045(05)70541-6

[4] Aoyama Y , Miyamoto A , Fujii T , Fujimori S , Tamaoka M , Takai D . Primary bronchial schwannoma: a case report. Medicine. 2022;101(40):e31062. 36221358 10.1097/MD.0000000000031062 PMC9542747

[5] Jin B , Wang T , Wang J , Qiu X , Pei Y , Wang Y , et al. Interventional bronchoscopic therapy in adult patients with tracheobronchial schwannoma. Ann Palliat Med. 2021;10(6):6279–6286. 34118848 10.21037/apm-21-630

